# Revisiting the in vivo GlnR-binding sites at the genome scale in *Bacillus subtilis*

**DOI:** 10.1186/s13104-017-2703-9

**Published:** 2017-08-23

**Authors:** Paola Randazzo, Anne Aucouturier, Olivier Delumeau, Sandrine Auger

**Affiliations:** Micalis Institute, INRA, AgroParisTech, Université Paris-Saclay, 78350 Jouy-en-Josas, France

**Keywords:** GlnR regulator, *B. subtilis*, ChIP-on-chip, Nitrogen metabolism

## Abstract

**Background:**

In *Bacillus subtilis*, two major transcriptional factors, GlnR and TnrA, are involved in a sophisticated network of adaptive responses to nitrogen availability. GlnR was reported to repress the transcription of the *glnRA*, *tnrA* and *ureABC* operons under conditions of excess nitrogen. As GlnR and TnrA regulators share the same DNA binding motifs, a genome-wide mapping of in vivo GlnR-binding sites was still needed to clearly define the set of GlnR/TnrA motifs directly bound by GlnR.

**Methods:**

We used chromatin immunoprecipitation coupled with hybridization to DNA tiling arrays (ChIP-on-chip) to identify the GlnR DNA-binding sites, in vivo, at the genome scale.

**Results:**

We provide evidence that GlnR binds reproducibly to 61 regions on the chromosome. Among those, 20 regions overlap the previously defined in vivo TnrA-binding sites. In combination with real-time in vivo transcriptional profiling using firefly luciferase, we identified the *alsT* gene as a new member of the GlnR regulon. Additionally, we characterized the GlnR secondary regulon, which is composed of promoter regions harboring a GlnR/TnrA box and bound by GlnR in vivo. However, the growth conditions revealing a GlnR-dependent regulation for this second category of genes are still unknown.

**Conclusions:**

Our findings show an extended overlap between the GlnR and TnrA in vivo binding sites. This could allow efficient and fine tuning of gene expression in response to nitrogen availability. GlnR appears to be part of complex transcriptional regulatory networks, which involves interactions between different regulatory proteins.

**Electronic supplementary material:**

The online version of this article (doi:10.1186/s13104-017-2703-9) contains supplementary material, which is available to authorized users.

## Background

The response of the Gram-positive bacterium *Bacillus subtilis* to nitrogen availability is an example of a highly sophisticated system to detect nitrogen levels and transmit this signal to effect intracellular enzyme activity and gene regulation. In this bacterium, ammonium assimilation occurs via the glutamine synthetase-glutamate synthase (GS-GOGAT) pathway to generate glutamate, the precursor for amino acids and nucleotides biosynthesis [1]. Glutamine is the *B. subtilis* preferred nitrogen source followed by arginine and ammonium [2, 3].

Two transcription factors, TnrA and GlnR, and one enzyme, the GS, play a major role in the *B. subtilis* nitrogen regulatory network [4–6]. TnrA and GlnR both control the expression of nitrogen-regulated genes with partial overlap of their respective regulon. They are active under different nutritional conditions. Under nitrogen-limited conditions of growth, TnrA acts on the transcription of a large regulon comprising at least 35 transcriptional units [7–13]. In particular, TnrA exerts an activating effect on the transcription of its own gene *tnrA* [4, 14] and represses that of *glnRA* and *gltAB* operons encoding GS and GOGAT, respectively [6, 15, 16]. On the contrary, in an excess of nitrogen, GlnR acts as a repressor of *tnrA*, *glnRA* and *ureABC* expression [4, 5, 17–19].

Glutamine acts as the metabolic signal for nitrogen availability. When glutamine is in excess it binds to and feedback inhibits GS by forming the complex FBI-GS that in turn directly interacts and sequesters TnrA, thus inhibiting its DNA-binding function [12, 20]. FBI-GS activates GlnR through a chaperoning interaction, which results in transcriptional repression of the *tnrA* and *glnRA* genes [5, 21–23].

TnrA binding sites have been defined as 17-bp inverted repeat sequences with the consensus TGTNANATTTTNTNACA [8, 13]. Indeed, GlnR and TnrA bind in vitro the same site upstream of the *tnrA* and the *glnRA* operon, albeit with different specificity [19]. It is proposed that the differences in GlnR and TnrA motifs appeared limited but large enough to bring about some specificity in their binding profile [24].

Despite knowledge of GlnR-regulated genes, a global identification of the TnrA/GlnR motifs directly bound by GlnR was still missing. Here, we used chromatin immunoprecipitation of GlnR-DNA complexes coupled with hybridization of DNA to tiled oligonucleotides arrays (ChIP-on-chip) to identify the GlnR DNA-binding sites in vivo, at the genome scale. We showed that GlnR binds efficiently 61 regions on the chromosome and overlaps partially the previously defined TnrA primary regulon [8]. Analysis with real-time in vivo transcriptional profiling allowed to show that GlnR represses expression of the TnrA-dependent *alsT* gene. Additionally, we characterized the GlnR secondary regulon, which is composed of promoter regions harboring a GlnR/TnrA box and bound by GlnR in vivo.

## Methods

### Bacterial strains and growth conditions

The *B. subtilis* strains used in this work are listed in Table 1. Luria–Bertani (LB) medium was used to cultivate *E. coli* and *B. subtilis*. *B. subtilis* cells were also grown in a modified Spizizen minimal medium containing 62 mM K_2_HPO_4_, 44 mM KH_2_PO_4_, 17 mM trisodium citrate, 11 mM K_2_SO_4_, 0.6% glycerol, 1 mM MgSO_4_, 1 mM CaCl_2_, 100 µM FeCl_3_ citrate, 112 µM ZnCl_2_, 5 µM MnCl_2_, 2.5 µM CuCl_2_, and 0.3% glutamate or 0.3% glutamine. When necessary, ampicillin, erythromycin, chloramphenicol, and spectinomycin were added at 100, 8, 5 and 100 µg ml^−1^, respectively. To obtain solid media, 20 g Agar noble l-1 (Difco) were added to the liquid media. To transform *E. coli* or *B. subtilis* cells, standard procedures were used as described in [25, 26].

**Table 1:** *Bacillus subtilis* strains used in this work

Strain	Genotype	Source
BSB1	*trp* ^+^	[32]
Bs005	*glnR*::*glnR*-*spa erm*	This study
Bs013	*perR*::*perR*-*spa erm*	This study
BSB21	Δ*glnR*::*spc*	This study
BSB53	Δ*tnrA*::*spc*	(Mirouze et al. [8])
BLUC85	P*alsT′*-*luc cat*	(Mirouze et al. [8])
BLUC86	P*alsT′*-*luc cat* Δ*tnrA*::*spc*	(Mirouze et al. [8])
BLUC302	P*alsT′*-*luc cat* Δ*glnR*::*spc*	This study
BLUC313	P*tnrA′*-*luc cat*	This study
BLUC314	P*tnrA′*-*luc cat* Δ*glnR*::*spc*	This study
BLUC315	P*tnrA′*-*luc cat glnR*::*glnR*-*spa erm*	This study

### DNA manipulations

DNA manipulations and cloning procedures were performed as described elsewhere [25]. DNA polymerase, restriction enzymes, and phage T4 DNA ligase were used according to the manufacturer’s instructions (Biolabs).

### Construction of a *glnR*::*glnR*-*spa* and *perR*::*perR*-*spa* strains

A *B. subtilis* strain was constructed to express a C-terminal SPA-tagged GlnR protein (hereafter GlnR^SPA^). A translational fusion between the *glnR* coding sequence and the sequential peptide affinity (SPA) tag sequence was integrated in the chromosome as described in [27, 28]. The pMUTIN-SPALIC vector (described by Doherty et al. [29]) was used to construct a pMUTIN-SPALIC derivative containing C-terminal SPA-tagged *glnR* gene. After transformation of wild-type BSB1 strain with this plasmid and selection for erythromycin-resistance, the strain Bs005 was obtained in which the expression of *glnR*-*spa* is under the control of the native *glnR* promoter, and the resulting GlnR^SPA^ is the only source of GlnR. The same strategy was used to construct the Bs013 strain expressing the PerR^SPA^ protein.

### Construction of Δ*glnR* deletion

The *glnR* mutant BSB21 was constructed by homologous replacement of the *glnR* coding sequence with the spectinomycin-resistance gene *spc* using a joining PCR technique [30]. Integration of the *spc* cassette at the *glnR* locus and deletion of the *glnR* gene were confirmed by DNA sequencing.

### Construction of luciferase promoter fusion strains

We used the strategy described previously in [8] by using the pUC18 cm-luc plasmid and the assembly Gibson’s procedure [31]. The primers used for PCR are indicated in Additional file 1: Table S2.

### Luciferase assay

We measured the luciferase activity as already described in details in [8] using a PerkinElmer Envision 2104 Multilabel Reader. Relative luminescence unit (RLU) and OD_600_ were measured at 5 min intervals.

### Genome-wide determination of the GlnR-binding sites by ChIP-on-chip

To measure the chromosome-wide DNA-binding profiles of GlnR, chromatin immunoprecipitation assays were performed as described previously [32]. The strain Bas005 was grown at 37 °C until an OD_600_ of 0.6 in minimal medium containing glutamine supplemented with 0.5 mM IPTG and 1 µg erythromycin ml^−1^. After cells treatment with formaldehyde, cellular DNA was extracted and sonicated. To purify the DNA regions specifically cross-linked to GlnR^SPA^ an antibody against the FLAG was used. The immuno-precipitated DNA (IP) and the control whole cell DNA extract (WCE) were labeled with Cy3 and Cy5, respectively, and co-hybridized to the *B. subtilis* Roche-NimbleGen tiled microarrays [33].

### Peak sequence extraction and analysis

To detect possible GlnR-binding sites from the chips, signal peaks were extracted, then the IP/WCE ratios (log2) were corrected and each peak was assigned a ChipScore as described in details in [34] and [35]. This score is based on the distribution of the peak height values and estimates for each peak its relative distance from the median. Only the regions associated with a peak scoring ≥4.0 in at least the two replicates were considered as putative GlnR-binding sites.

### SPA-tag pull-down experiments

The strains expressing the SPA fusions were grown to exponential phase in LB medium and the cells were recovered by centrifugation. Cells were frozen in liquid nitrogen. For tandem affinity purifications, cell pellets were resuspended with 5 ml of 10 mM Tris–Cl pH 8.0, 150 mM NaCl, 1 mg lysozyme ml^−1^, and 5 U Benzonase ml^−1^ (Novagen). Wild-type cells, which did not harbor a SPA fusion, were used as a control (no-SPA containing strain). GlnR^SPA^, PerR^SPA^ and No-SPA containing protein complexes were isolated and analyzed as described in [36].

## Results

### C-terminally SPA-tagged GlnR is a functional regulator

**Fig. 1:**
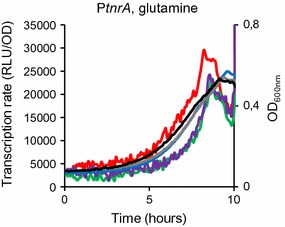
Expression of *tnrA* under the control of GlnR^WT^ and GlnR^SPA^. Promoter activity (RLU/OD) of a P*tnrA′*-*luc* transcriptional fusion with the *luc* reporter gene is indicated: purple line, wild-type; red line, Δ*glnR* cells; green line, *glnR*::*glnR*-*spa* cells. Strains were grown in minimal medium supplemented with glutamine as the sole nitrogen source. Growth (OD_600nm_) was monitored every 5 min: black lines, wild type; grey lines, Δ*glnR*; blue lines, *glnR*::*glnR*-*spa*. For each strain, one representative curve, out of three independent replicates realized, is shown

The *B. subtilis glnR* locus was modified to express the GlnR protein fused at its C-terminus with the SPA tag (GlnR^SPA^). In the resulting *glnR*::*glnR*-*spa* strain, the expression of the gene encoding the GlnR^SPA^ protein is under the control of its native transcriptional signals (see Methods section). To check the activity of the GlnR^SPA^ fusion protein, expression of the *tnrA* gene was compared in wild-type and *glnR*::*glnR*-*spa* strains. The expression of *tnrA* is known to be inhibited by GlnR [19]. The *tnrA* promoter region was fused with the *luc* reporter gene and introduced at the native *tnrA* locus in wild-type, *glnR*::*glnR*-*spa* and *glnR*::*spc* strains (Table 1). Light emission, which results from the activity of the *luc*-encoded firefly luciferase, was recorded every 5 min during growth in minimal medium with glutamine as sole nitrogen source. Expression of the *tnrA* promoter was repressed in the wild-type and *glnR*::*glnR*-*spa* strains whereas it was increased by a twofold factor in Δ*glnR* cells during the exponential growth phase (Fig. 1). We noticed that the transcription rate increased with time. This may be due to glutamine consumption from the medium in the used conditions. This entailed a decrease of GlnR repressive effect and an increase of TnrA activating effect on *tnrA* expression during the growth. Thus, GlnR^SPA^ was able to repress *tnrA* expression as GlnR^WT^. We concluded that the GlnR^SPA^ fusion protein was functional for transcriptional regulation.

### Genome-wide mapping of GlnR binding sites

**Fig. 2:**
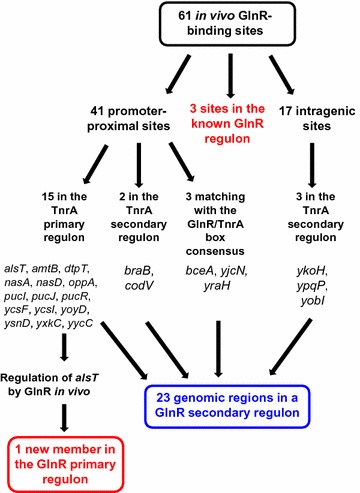
Analysis pipeline of the GlnR-binding sites detected by ChIP-on-chip. Several promoter regions associated to GlnR-binding sites are proposed to be classified in the two groups: GlnR primary (in *red*) and secondary (in *blue*) regulons

To identify GlnR-binding targets in *B. subtilis* genome, we carried out ChIP-on-chip experiments. The *glnR*::*glnR*-*spa* strain was grown in minimal medium with glutamine as the nitrogen source to exponential phase. After cross-linking, GlnR-bound DNA was immunoprecipitated using a FLAG specific antibody. Significantly GlnR-enriched DNA regions were identified as explained in the Methods section. Overall 61 enriched DNA regions were identified from the ChIP-on-chip signals (Additional file 2: Table S1). We retrieved GlnR-binding sites for the 3 well-characterized GlnR regulated promoters, *glnR*, *tnrA* and *ureA* (Fig. 2). In addition, 41 GlnR binding sites were detected less than 300 base-pairs upstream of a start codon. This suggests a GlnR-dependent expression of the nearest genes and therefore the existence of new candidates in the GlnR regulon. Finally, 17 peaks were located within intragenic regions more than 30 base-pairs downstream of a start codon (Fig. 2) (Additional file 2: Table S1). The location of these sites was intriguing since no GlnR intragenic binding sites have been described so far. It is possible that GlnR could bind to these intragenic sites to mediate repression by a roadblock mechanism, as described for the *B. subtilis* CcpA and CodY regulators [37, 38].

### GlnR-binding sites overlap the TnrA regulon

The GlnR and TnrA regulators are known to bind to DNA sites (GlnR/TnrA sites) that have similar pattern. Therefore, we compared the set of the newly identified GlnR-binding sites with the previously defined TnrA primary regulon [8, 13]. Fifteen of the GlnR-bound regions are located in TnrA-dependent promoter regions (Fig. 2) (Table 2). As one region is involved in the regulation of two divergent promoters (*nasA* and *nasB*) in total we recovered 16 well-characterized TnrA regulated promoters. In addition, 5 GlnR-binding sites overlapped the TnrA secondary regulon whose members are bound by TnrA in vivo but are not differentially regulated in a Δ*tnrA* strain [8]. These sites are located upstream of *braB* and *codV* translational start sites as well as in the encoding region of *ykoH*, *ypqP* and *yobI* (Additional file 2: Table S1).

**Table 2:** List of the genes located in the GlnR-binding regions detected by ChIP-on-Chip

Genes	Product
Common genes to GlnR and TnrA regulons	
*glnR*	Nitrogen sensing transcriptional regulator
*tnrA*	Nitrogen sensing transcriptional regulator
*ureA*	Urease
Genes in the TnrA primary regulon	
alsT*	Putative amino acid carrier protein; unknown
amtB*	Ammonium transporter
*dtpT*	Peptide transporter
*nasA*	Nitrate reductase
*nasD*	Assimilatory nitrite reductase subunit
*oppA*	Oligopeptide ABC transporter
pucI*	Allantoin permease
*pucJ*	Uric acid permease
pucR*	Transcriptional regulator of the purine degradation operon
*ycsF*	Putative nitrogen-containing heterocycle degradation enzyme
*ycsI*	Conserved hypothetical protein
*yoyD*	Putative exported protein
*ysnD*	Spore coat protein
*yxkC*	Unknown
*yycC*	Conserved hypothetical protein
Genes in the TnrA secondary regulon	
braB*	Branched-chain amino acid-Na+ symporter
codV*	Site-specific tyrosine recombinase
Genes containing a putative GlnR/TnrA box motif	
bceA*	Bacitracin ABC efflux transporter (ATP-binding protein)
yjcN*	Unknown
yraH*	Putative lyase
Other genes located in GlnR-binding regions	
*bdhA*	Acetoin reductase/2,3-butanediol dehydrogenase
*cotY;cotX*	Spore coat protein; spore coat protein
*dhbF*	Iderophore 2,3-dihydroxybenzoate-glycine-threonine trimeric ester bacillibactin synthetase
*gpsA*	NAD(P)H-dependent glycerol-3-phosphate dehydrogenase
*hmp*	Flavohemoglobin
*lysC*	Aspartokinase II alpha and beta subunit
*mmsA;iolR*	Methylmalonate-semialdehyde dehydrogenase; transcriptional repressor
*mntH*	Manganese transporter
*mutM*	Formamidopyrimidine-DNA glycosidase
*parA;yyaB*	Chromosome partitioning protein; putative membrane protein
*pksL*	Polyketide synthase of type I
*ppsA*	Plipastatin synthetase
*proS*	Prolyl-tRNA synthetase
*pucE*	Xanthine dehydrogenase, iron-sulfur subunit
*rghR*	Transcriptional repressor in sporulation initiation
*ylyB*	Similar to pseudouridylate synthase
*rocA*	Delta-1-pyrroline-5 carboxylate dehydrogenase
*rok*	Transcriptional repressor of genetic competence
*rasP*	Control of cell division, and SigV and SigW activity
*sinR*	Transcriptional regulator for post-exponential-phase response
*speE;speB*	Spermidine synthase; polyamine metabolism; agmatinase
*tyrS*	Tyrosyl-tRNA synthetase
*xlyB*	*N*-acetylmuramoyl-l-alanine amidase; bacteriophage PBSX protein
*ybxG*	Putative amino acid permease
*ycxD*	Putative transcriptional regulator
*yddJ*	Putative lipoprotein
*yddM*	Putative helicase
*yerO*	Putative transcriptional regulator
*yhdP*	Potential magnesium efflux pump
*yisK*	Putative catabolic enzyme
*yknU*	Putative ABC transporter (ATP-binding protein)
*ykoH*	Two-component sensor histidine kinase [YkoG]
*yktD*	Conserved hypothetical protein
*yobI*	Putative NTPase with transmembrane helices
*yobU*	Putative effector of transcriptional regulator
*yopQ*	Conserved hypothetical protein; phage Spbeta
*ypqP*	C-terminal part of the split gene spsM
*yrkK*	Putative integral inner membrane protein

In the ChIP-on-Chip experiments, 61 regions were detected as GlnR-binding targets. The GlnR-binding sites located near genes belonging to the GlnR and TnrA regulons are indicated. The asterisks indicate genes whose expression has been compared in the wild-type strain and in the Δ*glnR* mutant in this study

**Fig. 3:**
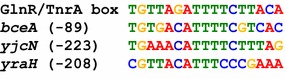
Identification of a 16-nt consensus sequence similar to the GlnR/TnrA motif in 3 in vivo GlnR binding sites. The 3 identified sequences are aligned with the previously reported GlnR/TnrA *box*

We further performed in silico analyses to investigate the presence of GlnR/TnrA boxes within the 38 newly identified inter- and intragenic GlnR-binding sites, which did not harbor a previously predicted GlnR/TnrA box. We used the MEME standard bioinformatic method [39] to identify common motifs among genomic regions representing 150 bp centered at each GlnR-binding site. We did not impose a constraint that the motif must be an inverted repeat sequence on the search. This yielded 16-nt sequences present in 3 GlnR-binding sites and matching the previously reported 17-nt TnrA box consensus with at least 10 identical nucleotides (Fig. 3) [8]. These potential GlnR/TnrA motifs are located in the promoter region of *bceA*, *yjcN* and *yraH* genes.

Half of the GlnR-binding sites detected by ChIP-on-chip did not display a significant match to the GlnR/TnrA box consensus. Using MEME, we were unable to identify a common DNA sequence motif among GlnR targets that lack a canonical GlnR/TnrA box motif. These suggest that GlnR recognizes degenerated GlnR/TnrA motif sequences, or that other factors are required for GlnR binding at these sites.

### In vivo GlnR-binding correlates with transcriptional regulation of the *alsT* gene

**Fig. 4:**
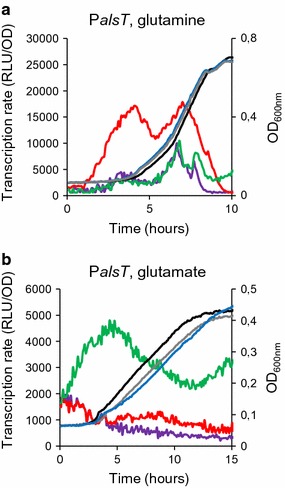
Expression of *alsT* under the dual control of GlnR and TnrA. Strains were grown in minimal medium supplemented with glutamate or glutamine as the sole nitrogen source. Growth (OD_600nm_) was monitored every 5 min: black lines, wild type; grey lines, Δ*glnR*; blue lines, Δ*tnrA*. **a** Promoter activity (RLU/OD) of a P*alsT′*-*luc* transcriptional fusion with the *luc* reporter gene is indicated: purple line, wild-type; red line, Δ*glnR* cells; green line, Δ*tnrA* cells. Strains were grown in the presence of glutamine. For each strain, one representative curve, out of three independent replicates realized, is shown. **b** Promoter activity (RLU/OD) of a P*alsT′*-*luc* transcriptional fusion with the *luc* reporter gene is indicated: purple line, wild-type; red line, Δ*glnR* cells; green line, Δ*tnrA* cells. Strains were grown in the presence of glutamate. For each strain, one representative curve, out of three independent replicates realized, is shown

We then tested the correlation between in vivo GlnR-binding and GlnR-dependent expression of the closer genes. Expression of 9 candidate genes containing a GlnR/TnrA box motif in their promoter region and covering the different groups that are illustrated on Fig. 2 was tested. We choose *alsT*, *amtB*, *pucI*, *pucR*, *braB*, *codV*, *bceA*, *yjcN* and *yraH* (Table 2). For this purpose, we used transcriptional fusions between the promoter regions and the luciferase gene in wild type and Δ*glnR* cells. Luciferase activity was recorded during exponential growth in minimal medium with glutamine as sole nitrogen source. In these conditions, transcription rate from P*alsT* was fourfold increased in a *glnR* mutant compared to wild-type (Fig. 4). As a control, expression of *alsT* was not altered in Δ*glnR* cells in the presence of glutamate as sole nitrogen source. In the glutamate-containing medium, *alsT* expression appeared repressed by both TnrA and GlnR in wild-type cells since *alsT* was derepressed in a *tnrA* mutant (Fig. 4) [13]. These results validated the GlnR-dependent regulation of the *alsT* gene. No difference in luciferase activity was observed for the 8 other gene fusions between wild type and Δ*glnR* strains in the conditions used (data not shown).

### GlnR^SPA^ is associated to the glutamate synthase and to TnrA in vivo

To provide insight putative interactions of GlnR with other transcriptional factors in vivo, we sought to identify GlnR^SPA^ binding partners. The strain expressing the *glnR*-*spa* fusion was grown in the nitrogen-rich LB medium in exponential phase. GlnR-associated proteins were purified and identified by mass spectrometry. Strains expressing no SPA-tagged protein and a SPA fusion to PerR, a non-related protein of *B. subtilis*, were used as negative controls [40, 41]. The TnrA and GltA proteins were specifically and reproducibly detected in the GlnR^SPA^ pull-down complexes (Table 3) based on the protein abundance index (PAI, established according to [42]. Therefore the GlnR^SPA^ protein is found in complex with the glutamate synthase and the TnrA regulator.

**Table 3:** GlnR is in complex with proteins TnrA and GltA

Protein partners	NO SPA	PerR-SPA	GlnR-SPA
GlnR	ND	ND	82
TnrA	ND	ND	4
GltA	1	ND	3

Protein partners eluted and quantified by LC–MS/MS in 3 independent SPA purification experiments using cells expressing TnrA-SPA or cells expressing no SPA-tagged protein (BSB1) or PerR-SPA as controls. Samples were taken in exponential growth phase. Numbers in the table correspond to the protein abundance index (PAI). Values are normalized to the total amount of peptides detected in each experiment

*ND* not detected

## Discussion

Using the ChIP-on-chip methodology, we have identified 61 enriched DNA-regions in the *B. subtilis* chromosome that are reproducibly bound by the GlnR regulator in abundant nitrogen growth conditions. As we recovered the known GlnR regulon, the whole GlnR binding sites identified by ChIP-on-chip could be considered as relevant. Our analyses revealed that a large overlap exists between the location of GlnR-binding sites and genes whose expression is regulated by TnrA. Fifteen GlnR-binding regions belong to the previously defined TnrA primary regulon (Fig. 1) [8]. Real-time in vivo transcriptional profiling enabled us to validate the repression of the *alsT* gene by GlnR in excess-nitrogen conditions (Fig. 3). Hence, *alsT* is submitted to a dual regulation by GlnR and TnrA, depending on the nutritional conditions. These data allow to define the GlnR primary regulon which is now composed of 4 transcription units (*glnRA*, *ureABC*, *tnrA* and *alsT*) fulfilling three criteria: (1) GlnR binding in ChIP-on-chip experiments; (2) the presence of a GlnR/TnrA box; (3) GlnR-dependent expression regulation.

Remarkably, 5 GlnR-binding sites are associated to regions reported to belong to the TnrA secondary regulon whose members are bound by TnrA in vivo but for which the conditions of a potential TnrA-dependent regulation are still unknown [8]. In addition, 3 GlnR-bound DNA regions correlates with the presence of in silico predicted GlnR/TnrA motifs (Fig. 3). Under conditions that maximize GlnR activity, expression of *braB*, *codV*, *bceA*, *yjcN* and *yraH* was similar in wild-type and *glnR* mutant cells. However, regulation of these genes is known to be driven by other transcription factors (Additional file 2: Table S1). Therefore the existence of complex regulatory networks could mask GlnR activity.

Altogether, the ChIP-on-chip approach allowed us to define a GlnR secondary regulon, which is composed of 23 genomic regions fulfilling two criteria: (1) in vivo GlnR binding in ChIP-on-Chip experiments; (2) the presence of a GlnR/TnrA motif. We propose that GlnR might play a regulatory role in specific unknown conditions. The composition of the secondary regulon cannot be clearly delimited and is opened to permutations with the primary regulon depending on the discovery of yet unknown conditions involving GlnR-dependent regulation. We assume that expression of some genes could respond to specific growth conditions leading to intermediate levels of GlnR activity, as exemplified by the regulation of *braB* by the CodY regulator [43]. Moreover, we observed that GlnR belong to a protein complex in vivo with the glutamate synthase GltA. The potential role of a direct interaction between GlnR and GltA in the control of transcriptional regulation deciphers further investigations.

Finally, we reported a set of 35 GlnR binding DNA sites, which did not harbor a canonical GlnR-binding motif, suggesting that GlnR recognizes degenerated GlnR box sequences or that other factors are required for GlnR binding at these sites. It was previously shown that a GlnR protein truncated in the C-terminal domain repressed more tightly the expression of its target genes than the wild-type GlnR [44]. Deletions in the C-terminal region of GlnR [44] or TnrA [45–47] abolished their interaction with GS. Thus, we cannot exclude that addition of a SPA tag in the C-terminal part of GlnR might have changed its binding affinity to DNA as well as the interaction specificity with GS and the regulatory control. This could also explain that GS was not detected as protein partner in the GlnR^SPA^ pull-down complexes.

The binding characterization of GlnR to DNA regions without evident GlnR-binding motif would be an important improvement to understand the role of GlnR and require further studies. *In vitro* assays could be performed to study the direct interaction between the native GlnR protein and the DNA regions that do not have a GlnR-binding motif. However, the binding of GlnR to these sites might require other unknown regulatory factors or specific conditions. It will be necessary to develop in vivo approaches to study the binding of GlnR to the newly identified targets and the consequences on the regulation of the nearest genes. Moreover, the surprising interaction detected between GnR and GltA deserve further investigations.

## Conclusions

In the light of our results, we propose that binding of GlnR and TnrA to the same DNA binding sites may allow fine control over gene expression in response to various nitrogen levels. GlnR appears to be a part of complex transcriptional regulatory networks, which involves interactions between different regulatory proteins. In vivo, GlnR is found in complex with the GltA and TnrA proteins. Further investigations are required to define the exact role of the GlnR regulator in the control of the newly identified in vivo binding sites.

## Additional files

**Additional file 1: Table S2.** Oligonucleotide primers used in this study.

**Additional file 2: Table S1.** Mapping of GlnR DNA binding sites by ChIP-on-chip. ChIP-on-chip experiments were performed and data were analysed as previously described [32] using the method described by Reppas et al. [34]. GlnR was purified in two biological replicates for each condition of growth. This table lists all significantly enriched DNA regions by ChIP-on-chip experiment performed with a GlnR^SPA^ expressing *Bacillus subtilis* strain.

## Abbreviations

ChIP-on-chip: chromatin immunoprecipitation coupled with hybridization to tiled oligonucleotides arrays
GS: glutamine synthetase
bp: base pair
SPA tag: sequential peptide affinity tag
OD: optical density
RLU: relative luminescence unit
PCR: polymerase chain reaction
