# Supplementary material for: Revisiting the in vivo GlnR-binding sites at the genome scale in Bacillus subtilis
Source: BMC Res Notes. 2017 Aug 23;10:422. doi: 10.1186/s13104-017-2703-9 (PMC5569456; doi:10.1186/s13104-017-2703-9)
Supplement: Supplementary file 1 — Additional file 1: Table S2. Oligonucleotide primers used in this study. [file 13104_2017_2703_MOESM1_ESM.doc]

**Table S2 Oligonucleotide primers used in this study**

| **Name** | **Sequence (5'→3')** |
| --- | --- |
| F- pUC18-luc | CTCTAGAGGATCCCCGGGTACCAG |
| R- pUC18-luc | TCGACCTGCAGGCATGCAAGCTTG |
| F-tnrA | **CAAGCTTGCATGCCTGCAGGTCGA**CAAGCAGAAGTCTCGACCCA |
| R-tnrA | **CTGGTACCCGGGGATCCTCTAGAG**CCCCTGGATGTCTTTTGAT |
| F-alsT | **CAAGCTTGCATGCCTGCAGGTCGA**TGGTTGGTGA ATAGATCTGTTAC |
| R-alsT | **CTGGTACCCGGGGATCCTCTAGAGC**ATCAATTCAATACGAATTCGATT |
